# Expression of Toll-like receptors (TLRs) in the lungs of an experimental sepsis mouse model

**DOI:** 10.1371/journal.pone.0188050

**Published:** 2017-11-14

**Authors:** Anargyros Bakopoulos, Alkistis Kapelouzou, Diamantis I. Tsilimigras, Michalis Katsimpoulas, Dimitrios Schizas, Chrysostomos Aravanis, Evaggelos Balafas, Manolis Mavroidis, Kitty Pavlakis, Anastasios Machairas, Theodore Liakakos

**Affiliations:** 1 Third Department of Surgery, Attikon General Hospital, School of Medicine, National and Kapodistrian University of Athens, Athens, Greece; 2 Center for Clinical, Experimental Surgery and Translational Research, Biomedical Research Foundation of the Academy of Athens, Athens, Greece; 3 School of Medicine, National and Kapodistrian University of Athens, Athens, Greece; 4 1st Department of Surgery, Laikon General Hospital, School of Medicine, National and Kapodistrian University of Athens, Athens, Greece; 5 Laboratory Animal Facilities, Biomedical Research Foundation of the Academy of Athens, Athens, Greece; 6 Basic Research, Biomedical Research Foundation of the Academy of Athens, Athens, Greece; 7 Department of Pathology, School of Medicine, University of Athens, Athens, Greece; University of Szeged, HUNGARY

## Abstract

**Background:**

Sepsis is a condition characterized by high mortality rates and often accompanied by multiple-organ dysfunction. During sepsis, respiratory system may be affected and possibly result in acute respiratory distress syndrome (ARDS). Toll-like receptors (TLRs), as a first line defense against invading pathogens, seem to be highly expressed in septic states. Therefore, expression of TLRs in the lungs of a sepsis animal model could indicate the involvement of the respiratory system and appear as a severity index of the clinical course.

**Materials and methods:**

A total of 72 C57BL/6J mice, aged 12–14 weeks, were studied. The animals were divided into 3 sepsis (S) groups (24h, 48h and 72h) and 3 control (C) groups (24h, 48h and 72h), each consisting of 12 mice. The S-groups were subjected to cecal ligation and puncture (CLP) while the C-groups had a sham operation performed. Blood samples were drawn from all groups. Total blood count analysis was performed along with the measurement of certain biochemical markers. Additionally, lung tissues were harvested and the expression of TLRs, namely TLR 2, TLR 3, TLR 4 and TLR 7 were evaluated by means of immunofluorescence (IF) and qRT-PCR (quantitative-Polymerase Chain Reaction). Statistical analysis was performed by using one-way ANOVA followed by student t-test. Results were considered statistically significant when p<0.05.

**Results:**

WBCs and lymphocytes were decreased in all S-groups compared to the corresponding C-groups (p<0.05), while RBCs showed a gradual decline in S-groups with the lowest levels appearing in the S72 group. Only, monocytes were higher in S-groups, especially between S48-C48 (p<0.05) and S72-C72 (p<0.05). Creatinine, IL-10 and IL-6 levels were significantly increased in the S-groups compared to the corresponding C-groups (S24 vs C24, S48 vs C48 and S72 vs C72, p<0.05). IF showed that expression of TLRs 2, 3, 4 and 7 was increased in all S-groups compared to the time-adjusted C-groups (p<0.05). Similarly, qRT-PCR revealed that expression of all TLRs was higher in all S-groups compared to their respective C-groups in both lungs and intestine (p<0.05). Comparing lung and intestinal tissues from S-groups, TLRs 2 and 4 were found increased in the lung at 24, 48 and 72 hours (p<0.05), whereas TLR 3 was higher in the intestine at all time points examined (p<0.05). Finally, TLR 7 levels were significantly higher in the intestinal tissues at 24 hours (p<0.0001), while lungs predominated at 48 hours (p<0.0001).

**Conclusion:**

TLRs seem to be highly expressed in the lungs of septic mice, therefore suggesting a potential role in the pathogenesis of ARDS during sepsis. While more studies need to be conducted in order to completely understand the underlying mechanisms, TLRs may represent a promising target for establishing novel therapeutic strategies in the treatment of sepsis.

## Introduction

Sepsis constitutes a fatal syndrome caused by a dysregulated host response to infection [1]. Numerous studies show that in the setting of intensive care unit (ICU), the incidence of sepsis and its related mortality may reach 12% and 40%, respectively [2]. Nowadays, sepsis affects millions of people recording constantly increasing incidence rates every year. Unfortunately, more than 25% of septic patients seem to finally succumb [3–7]. Several mediators such as cytokines, chemokines, complement-activating products and lately Toll-like receptors (TLRs) have been identified in the process of sepsis, each involved in distinct or common pathways that collectively lead to the establishment of this fatal state [8]. Elucidating these complex underlying mechanisms is, therefore, rendered crucial so as to shed light on the development of innovative therapeutic strategies.

Due to the inherent difficulties in studying these mechanisms in humans, several efforts have been made to create animal models that closely resemble septic states. Amongst them, cecal ligation and puncture model (CLP) is considered by many researchers as the gold standard technique for inducing sepsis [9]. It is noteworthy that CLP produces a polymicrobial infection with resultant hemodynamic and biochemical responses that are equivalent to those in humans [9–11].

TLRs belong to a family of pattern-recognition receptors (PRPs) that initiate certain patterns of host defense after recognizing either tissue damage or microbial infections [8]. Upon activation, they mainly trigger the innate immune response, thus culminating in the production of proinflammatory cytokines [12]. To date, ten TLRs have been discovered in humans while thirteen in mice [13], with TLR 4 and TLR 2 being the most widely investigated molecules overall [14]. The discovery of TLRs has aided in the understanding of the molecular pathways between innate immunity, inflammation, and a wide variety of diseases. At present, they are considered crucial receptors for the initiation of the inflammatory response in sepsis [12,15].

The respiratory system is a well-known target in sepsis. Due to the systemic inflammation, lungs are characterized by profound cell infiltration, non-cardiogenic pulmonary edema, and diffuse alveolar damage that eventually leads to respiratory failure [16,17]. Acute lung injury (ALI) and acute respiratory distress syndrome (ARDS) are both life-threatening conditions that may appear in the setting of a septic state [16]. However, the mechanistic role of bacterial peritonitis-induced sepsis in lung pathology has remained elusive. Interestingly, TLRs are widely expressed on lung cells and specifically, TLR 4 has proved a key component in a pathway that controls the severity of acute lung injury (ALI) [17]. We, therefore, tried to determine the role of TLRs 2, 3, 4 and 7 in a well-established sepsis mouse model and associate the expression of TLRs with the severity of sepsis.

## Material and methods

### A. Animal model

In total, we sacrificed 72 male C57BL/6J mice, aged 12–14 weeks, supplied from the colony of the Centre of Experimental Surgery of the Biomedical Research Foundation of the Academy of Athens. The study protocol was approved by the local ethics committee (Athens Prefecture Veterinarian Service; K/2953/23-4-2007) and took place in the animal facilities of the Center of Experimental Surgery of our institution (EL 25 BIO 003). All the methods applied regarding the care and the handling of the animals were carried out in accordance with the internationally approved guidelines. More specifically, animals were kept in separate cages under stable conditions (temperature 18–21°C, humidity 40–50%, artificial day-night cycle of 12:12 hours) with free access to water and chow using standardized and balanced industrial nutrition. During the study, the body weight of all animals was regularly measured (20-25gr), in order to control the septic animal status and prevent any adverse reaction that could possibly result in biased results.

### B. Experimental procedure/Protocol

The animals were initially randomized into 6 groups, with three of them comprising the sepsis (S) groups. Regarding these groups, cecal ligation and puncture (CLP) was performed under isoflurane-induced anaesthesia (induction with 5% and maintenance with 3.5–4.5% in 0.5lt/min O_2_ flow). All interventions were executed in line with a previous published protocol [18]. At first, the abdominal area was shaved and disinfected by applying an antiseptic solution (Betadine, Mudipharma S.A, Switzerland). A 1cm midline abdominal incision was made and the cecum was exposed with careful maintenance of its vascular supply. The distal one-third of the cecum was later ligated with a 3.0 Silk suture (Johnson and Johnson, Edinburgh, UK) and was once punctured through with a 21-gauge needle allowing the release of fecal material into the peritoneal cavity. Finally, the cecum was placed back into the peritoneal cavity and the incision was closed in two layers with a 4.0 Vicryl suture (Johnson and Johnson, Edinburgh, UK). In the piloting setting, we observed that one puncture was enough to induce peritonitis and sepsis, whereas more penetrations resulted in early death, sometimes within 24 hours of onset, thus not allowing the completion of the protocol. The severity of sepsis following CLP procedure was assessed based on a previous published scoring system [19]. If the body condition reached values >10, the mice were to be sacrificed and the experiment terminated [19].

The other three groups were used as controls (C groups). A similar operation was performed except for ligation and puncture of the cecum (sham operation). No fecal material was seen in the peritoneal cavity. The aforementioned operation was executed so as to represent the operation stress in the control groups, thus allowing for more reliable results. The postoperative analgesic protocol consisted of repeated injections of buprenorphine (0.05mg per kg) subcutaneously every 6 hours for at least 2 days in both septic and control groups. All animals were resuscitated with 1ml of subcutaneously-administered prewarmed (37°C) isotonic sodium chloride solution. Mice were returned to cages in a temperature-controlled room (22°C) immediately at the end of the surgical procedures where access to water and food was available and postoperative monitoring was performed every half hour for at least 2 hours.

Animals in S-groups were sacrificed at 24h, 48h and 72h after the operation (S24, S48 and S72 groups) with the animals in the C-groups being sacrificed at the same time points (C24, C48 and C72 groups) following the sham operation that was previously described. Euthanasia was achieved with induction of anesthesia using isoflurane -as described above- and exsanguination by cardiac puncture. Body weight was measured before euthanasia and blood samples were drawn by intracardiac aspiration. Finally, intestine and lung tissues were harvested and placed in 10% formaldehyde for further tissue analysis, or in -80C for qRT-PCR analysis.

### C. Blood analysis

Depending on the time of euthanasia of each group, blood samples were taken at 24, 48 and 72 hours following the operation. Parameters that were extracted and analyzed are as follows: white blood cell (WBC) and red blood cell (RBC) concentration, lymphocytes, monocytes, creatinine, IL-6 and IL-10. All serum samples were measured in duplicate and were analyzed in blood test analyzer machine (Nihon Kohden, Japan); IL-6 and IL-10 were determined using commercially available Elisa kits (Quantikine mouse IL-6, IL-10 immunoassay kit; R&D systems, Wiesbaden, Germany) following the instruction of the manufacturer; finally creatinine was analyzed in biochemical machine (chemical 2910 Awareness technology Inc, FL, U.S.A) by enzymatic colorimetric methods using commercial kits (Human, Germany).

### D. Tissue preparation

After euthanasia, the lungs of each mouse were harvested and subsequently perfused with ice-cold phosphate buffer solution (PBS), pH 7,4. Each organ obtained was further divided into two separate pieces. One part was fixed in formalin and embedded in paraffin blocks while the other was prepared in diethyl pyrocarbonate (DEPC) treated solution and immediately stored at a temperature of -80°C for subsequent mRNA analysis. Intestinal tissues were also stored at -80°C for PCR analysis.

### E. Histology—Immunofluorescence

#### Histology

H&E staining was performed in ten paraffin sections (5μm) to determine the tissue damage between S- and C-groups on cellular level.

#### Immunofluorescence antibodies

The following rabbit polyclonal antibodies were used: (a) TLR 2 sc10739; b) TLR 3 sc28999; c) TLR 4 sc30002; d) TLR 7 sc30004. A specific secondary goat anti-rabbit antibody TRITC-sc 2780 used for the detection of primary antibodies.

#### Method

Immunofluorescence was performed on paraffin-embedded sections. Unmasking of the antigen retrieval was performed by heat-mediated antigen retrieval method in 0.01M citric acid (pH 6.0). The ZytoChem Plus HRP kit Broad spectrum (#HRP060, ZYTOMED Systems GmbH, Germany) was employed according to the manufacturer’s instructions.

In brief, 5 μm paraffin sections were deparaffinized at 60°C, immersed in xylene and hydrated in a graded series of ethanol aqueous solutions. Antigen retrieval was performed by using a citrate buffer (0.01M, pH 6.0) and heating the sample for 10 minutes with an 800 W microwave. Washes were performed with Phosphate buffer solution following each step of the protocol. Sections then were immersed in freshly prepared 3% hydrogen peroxide (H_2_O_2_) for 10 minutes in the dark, blocking endogenous peroxidase activity. Avidin/Biotin complex solution was immersed for 15 mins in room temperature. Then sections were incubated with the blocking serum supplied by the ZytoMed (Plus HRP kit, Broad Spectrum kit; Berlin, Germany) for 5 minutes to block non-specific staining. Antibodies against TLR 2, 3, 4 and 7 were diluted in 1:200 in 0.01 mol/l phosphate-buffered saline (PBS), pH 7.4, and finally were incubated at 4°C overnight. Sections were washed 3 times for 5 minutes with PBS. A diluted (1:200) specific secondary goat anti-rabbit antibody TRITC was incubated for 60 minutes at room temperature. Sections were washed 3 times for 5 minutes with PBS. DAPI counterstaining was used for the nuclei. Aqueous medium was used for the coverslip. The immunofluorescence sections were examined by microscopic analysis in Leica DMRA2 microsystem (Leica microsystems, USA) by using digital camera C11440 ORCA flash 4.0 (HAMAMMATSU, JAPAN).

#### Morphometry

Morphometry was performed to measure the area of each tissue, the percentages of the background, cellular nuclei and expression of each TLR using the Image J program (version 1.49C, Wayne Rasband, National Institutes of Health, USA) to analyze every image. For the measurement of the relative concentrations of the stained molecules, the segmental stained tissue area was expressed as percentage of the whole tissue area.

### F. Quantitative Real-Time PCR (qRT-PCR)

Total RNA was extracted using the Trizol reagent according to the manufacturer’s protocol (Life Technologies-Invitrogen). 1 μg of total RNA was used to perform reverse transcription and cDNA was generated using the MMLV reverse transcriptase. Primers used for the PCR were produced by Integrated DNA Technologies (Leuven, Belgium) (S1 Table). qRT-PCR was performed using the LightCycler 480 (Roche Mannheim, Germany). Briefly, each 20μl reaction contained 2μl cDNA (20ng of total RNA), each primer at 200 nM and 10 μl of Kapa Sybr Fast qPCR master mix (KAPA BIO, Boston MA, USA). After an initial denaturation step at 95°C for 10 min, the PCR conditions were: 95°C×30s, 60°C×40 s, 72°C×40s, 40 cycles. All samples were repeated in duplicate and the mean Ct value for each sample was used for data analysis. The 2-ΔΔCT method analysis of relative gene expression using qRT-PCR was used to calculate the relative changes in gene expression. All data were normalized by GAPDH levels and expressed as % relative to controls, as previously described [20].

### G. Statistical analysis

Results were analyzed using the one-way ANOVA followed by student t-test. Differences were considered as statistically significant if the null hypothesis could be rejected with > 95% confidence (p < 0.05). The statistical data analysis was performed using the Graph Pad Prism program version 4.03.

## Results

### Hematological and biochemical analysis

As shown in Table 1, WBCs and lymphocytes were decreased in S-groups (S24, S48, S72) compared to the corresponding C-groups (C24, C48, C72, respectively) (p<0.05). However, there was a significant increase among S-groups as time to euthanasia was longer; S72 versus S48, S72 versus S24 and S48 versus S24 (p<0.05). On the other hand, RBCs showed a gradual decline in S-groups with the lowest levels appearing in the S72 group. Only monocytes were higher in S-groups, especially between S48-C48 (p<0.05) and S72-C72 (p<0.05), whereas a significant increase was observed among S-groups: S72 versus S48, S72 versus S24 and S48 versus S24 (p<0.05).

**Table 1:** Blood and biochemical results.

Time groups			
Groups	24h	48h	72h
	(n = 24*)	(n = 24*)	(n = 24*)
**WBC (10^3/μL)**			
Control	7.4±1.6^a^	7.64±1.77^a^	7.66±1.78^a^
Septic	2.28±0.38	3.48±0.23^b^	5.37±0.31^c^^,^ ^d^
**RBC (10^6/μL)**			
Control	7.23±1.54	7.25±1.44^a^	7.31±1.28^a^
Septic	6.13±1.67	5.0±1.86	2.85±0.72^c^^,^ ^d^
**Lymphocytes (%)**			
Control	78.67±10.01^a^	80.33±10.86^a^	79±9.12^a^
Septic	62.67±4.59	54.83±2.04^b^	35.67±2.94^c^^,^ ^d^
**Monocytes (%)**			
Control	3.5±1.64	3.67±1.63^a^	3.83±1.47^a^
Septic	3.5±1.76	5.67±0.82^b^	7.33±0.51^c^^,^ ^d^
**Creatinine (mg/dL)**			
Control	0.36±0.15^a^	0.47±0.17^a^	0.38±0.16^a^
Septic	1.52±0.28	2.47±0.31^b^	3.48±0.77^c^^,^ ^d^
**IL-6 (pg/ml)**			
Control	13.04±3.6^a^	12.93±2.77^a^	14.93±3.12^a^
Septic	1408±328.5	2306±432.6^b^	3036±893.8^c^^,^ ^d^
**IL-10 (pg/ml)**			
Control	7.44±1.59^a^	7.58±1.22^a^	7.72±1.22^a^
Septic	100.9±13.65	196.5±35.14^b^	1182±605.1^c^^,^ ^d^

Values are expressed as mean ± SD. Statistical significances (p<0.05) between the groups at the same time point are indicated as follows:

^a)^ Control vs Septic;

^b)^ 24S vs 48S,

^c)^ 48S vs 72S,

^d)^ 24S vs 72S

* At each time point (24h, 48h and 72h), septic (n = 12) and control (n = 12) groups were compared in terms of blood and biochemical parameters.

Biochemical analysis revealed that creatinine, IL-10 and IL-6 were significantly increased in the S-groups (S24, S48, S72) compared to the corresponding C-groups (C24, C48, C72) (p<0.05). Among S-groups, creatinine and IL-10 reached the highest levels in S72 group while IL-6 in the S48, though not significantly higher than S72 group.

### Histology and immunofluorescence

**Fig 1:**
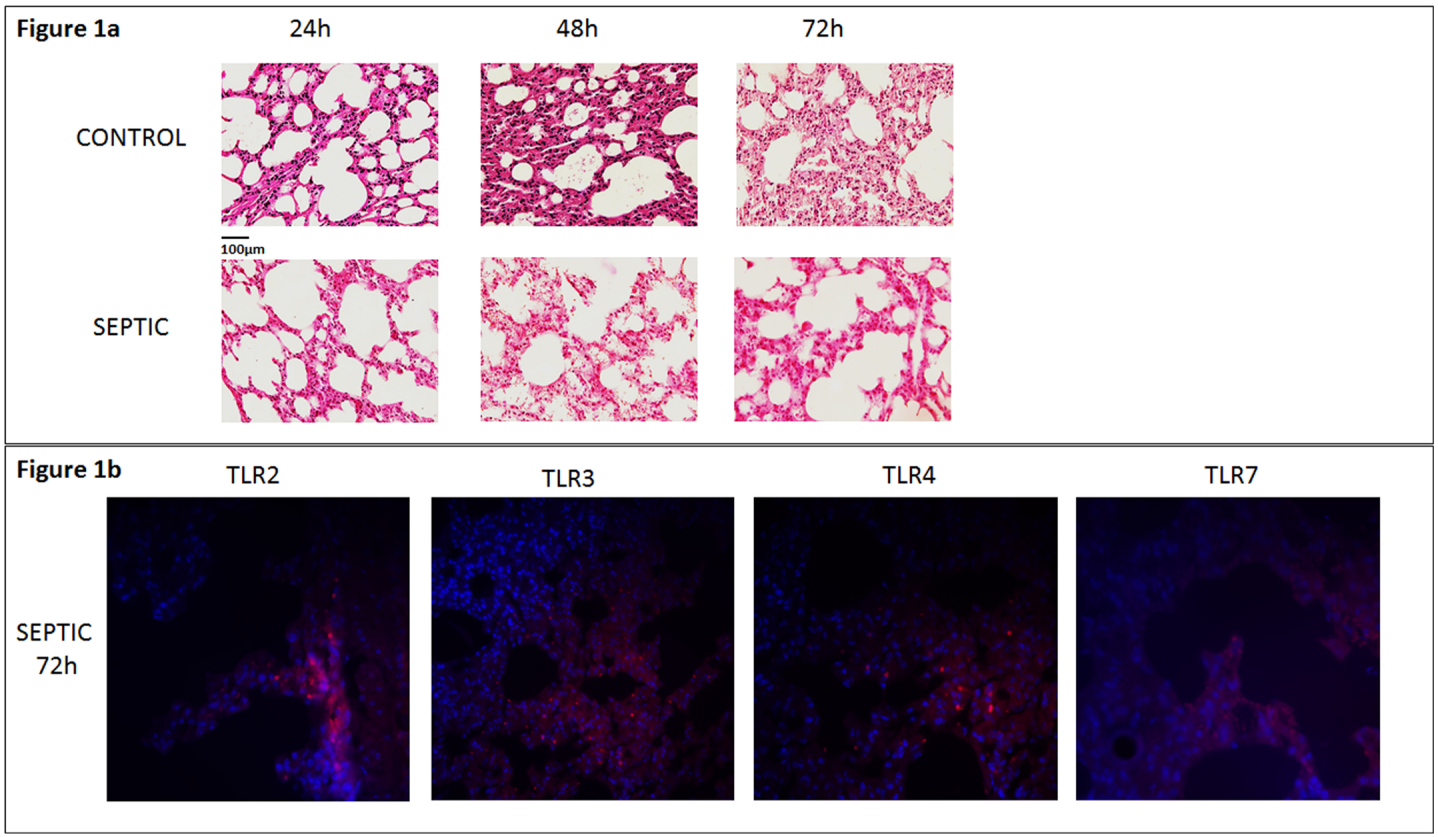
**a)** Lung tissue sections are stained with hematoxylin and eosin. Images (x40) from lung sections are shown at 24h, 48h and 72h after sham operation or CLP procedure. Control mice (sham operated), (upper panel in 1a) and septic mice (down panel in 1a) demonstrate no lung injury and increased lung injury observed during time period, respectively. Different degrees of acute lung injury assessed by histological examination in septic mice. Observed signs of edema and alveolar damage detected from 24 h after CLP, including alveolar flooding and alveolar collapse. **b)** Immunofluorescence staining expressing TLR 2, TLR 3, TLR 4 and TLR 7 in capillaries at 72h after CLP challenge. Among septic mice, the highest expression of TLRs was noted at this time point (down panel). TLR-positive cells in the lung of septic mice were stained red, while cell nuclei were stained blue. Original magnification x63.

**Fig 2:**
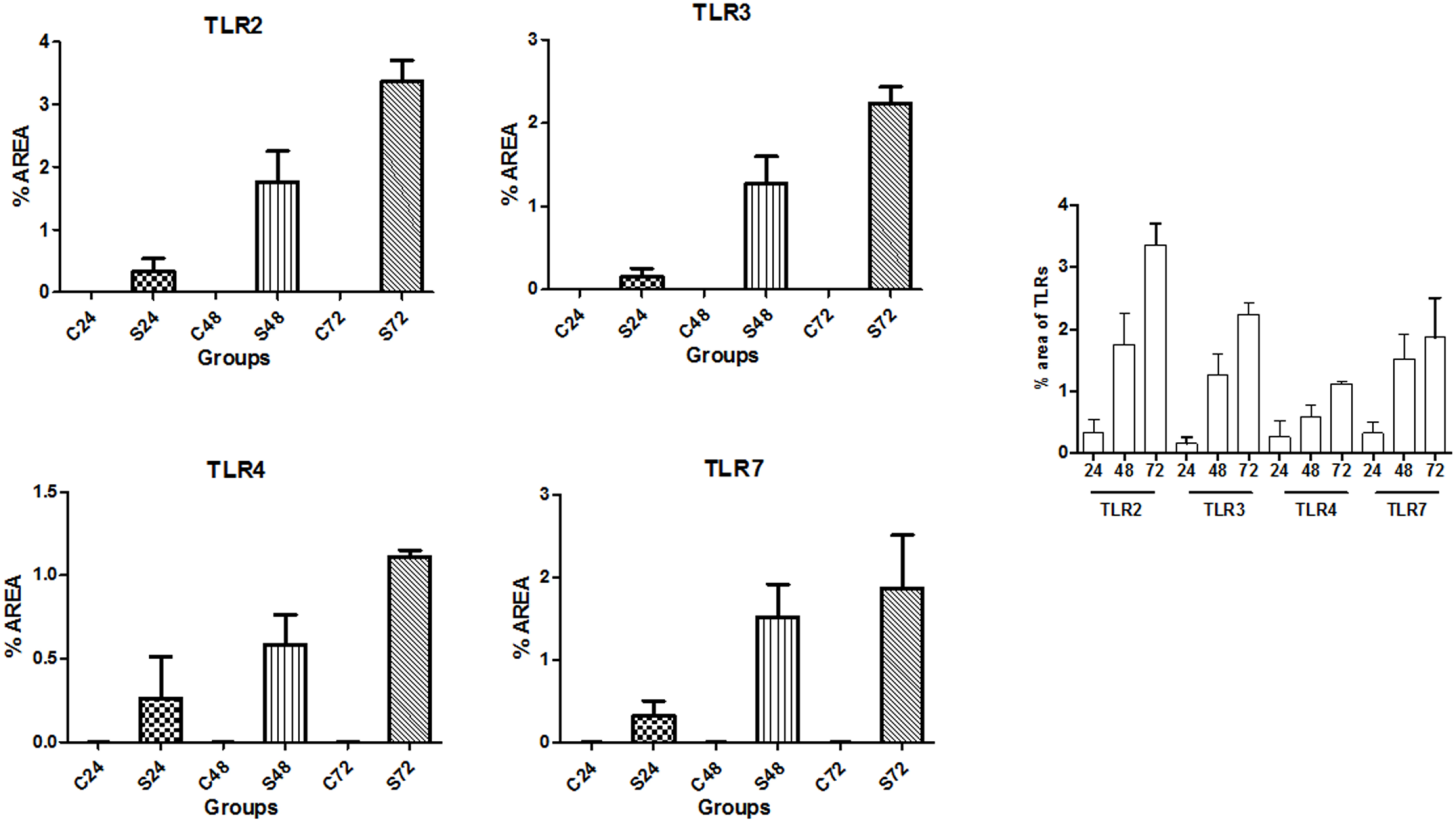
**a-d)** Analysis of lung tissues through IF showed a significantly increased area (%) of expression of TLRs 2, 3, 4 and 7 in all S-groups compared to the time-adjusted C-groups (p<0.05), **e)** Among septic mice, expression of TLRs 2, 3 and 4 was higher in S48 versus S24 groups (p<0.05), S72 versus S24 groups (p<0.05) and S72 versus S48 groups whereas expression of TLR 7 was higher in S48 versus S24 groups (p<0.05), S72 versus S24 groups (p<0.05), but yet comparable between S72 and S48 groups (p>0.05).

Lung tissues from each group were analyzed by means of IF and expression of TLR 2, 3, 4 and 7 was quantitatively examined. Representative images of histology and IF are shown in Fig 1a and 1b. Analysis of lung tissues through IF showed a significantly increased area (%) of expression of TLRs 2, 3, 4 and 7 in all S-groups compared to the time-adjusted C-groups (p<0.05) (Fig 2a, 2b, 2c, 2d and 2e). In addition, expression of every TLR examined was higher in S48 versus S24 groups (p<0.05) as well as in S72 versus S24 groups (p<0.05). Finally, significant increase exhibited in S72 compared to S48 groups, regarding the expression of TLR 2, 3 and 4 (p<0.05). No significant difference was noticed between S72 and S48 groups with regards to TLR 7. Further details on the expression of each TLR (mean ±SD) in both C- and S-groups are provided in the S2 Table.

### qRT-PCR

**Fig 3:**
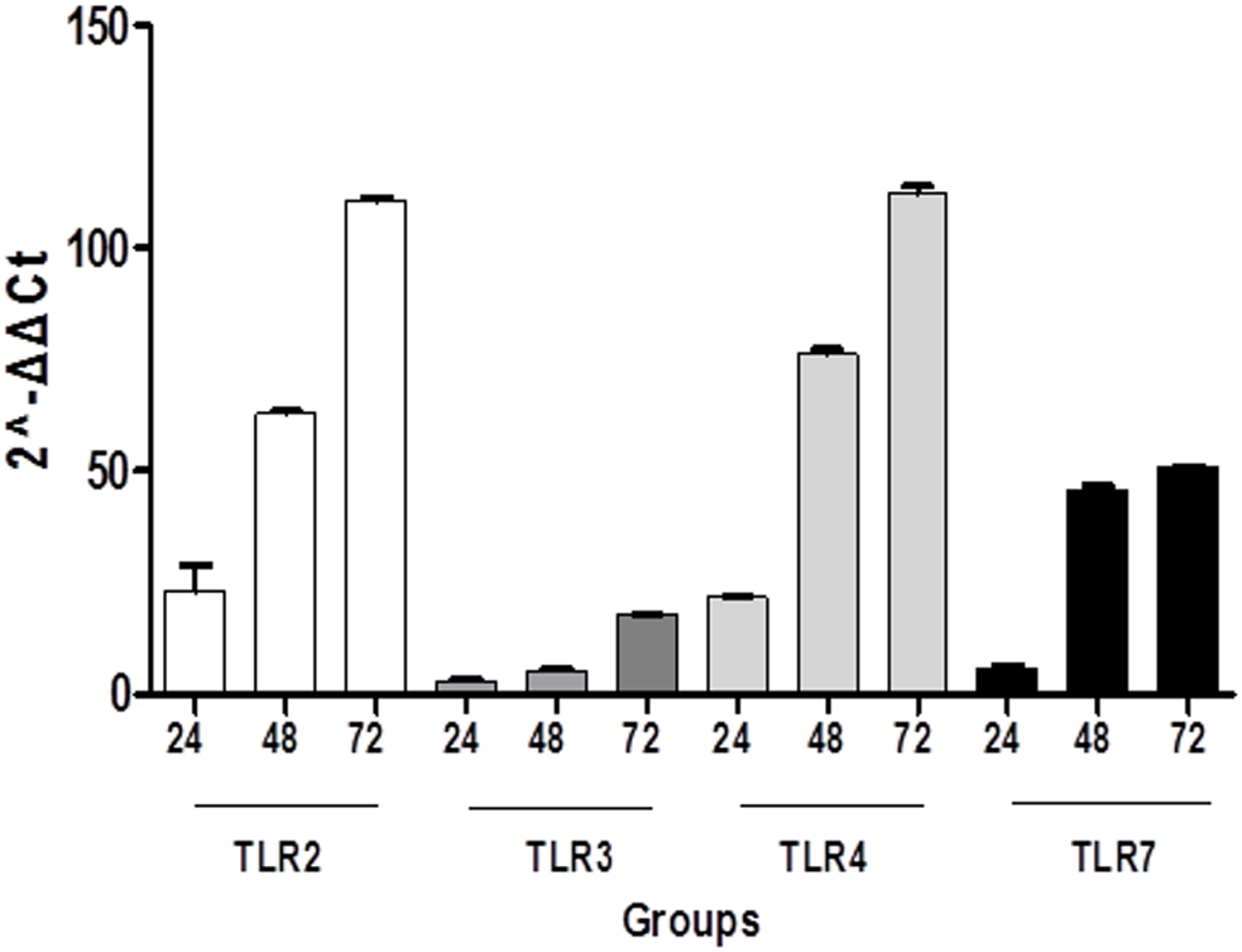
Analysis of lung tissues through qRT-PCR showed S-groups yielded a significantly higher expression of all TLRs in S-groups compared to their time-adjusted C-groups (p<0.05). The most significant variances were observed between S72-C72 followed by S48-C48 groups concerning TLR 2 and TLR 4, whereas the least important differences were noticed between S24-C24 regarding TLR 7 and TLR 3.

Apart from IF results, qRT-PCR was employed on mouse lung tissues as well. It was shown that S-groups yielded a significantly higher expression of all TLRs under investigation compared to their respective C-groups (p<0.05). The most significant variances were observed between S72-C72 followed by S48-C48 groups concerning TLR 2 and TLR 4, whereas the least important differences were noticed between S24-C24 regarding TLR 7 and TL 3 (Fig 3). Another interesting finding is that among S-groups, mRNA expression of each TLR was higher in S72 followed by S48 and finally S24 groups reaching a statistically significant level when compared to each other (S48 versus S24, S72 versus S48 and S72 versus S24) (S3 Table).

**Fig 4:**
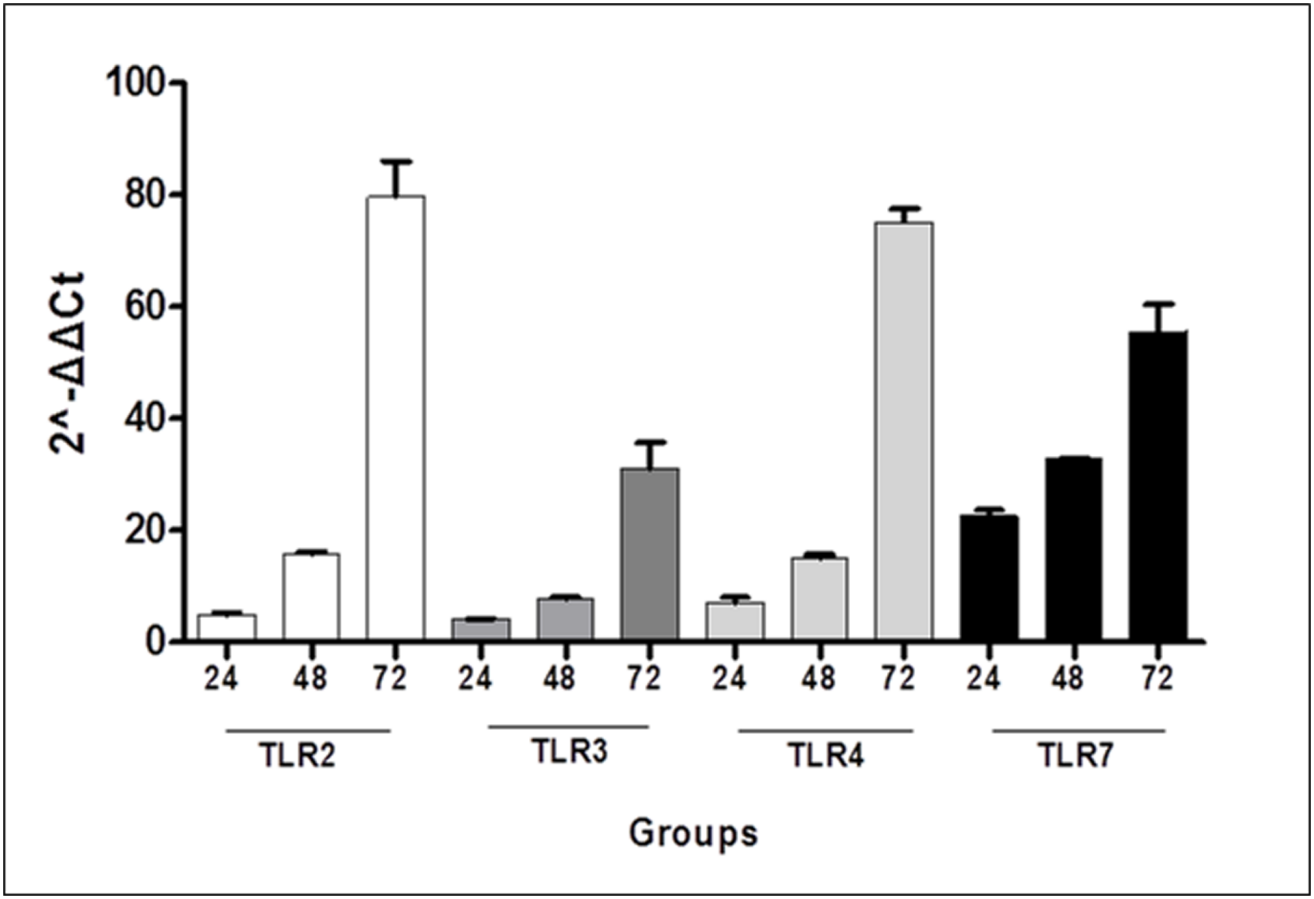
Among S-groups, qRT-PCR of intestinal tissues showed significant differences between S48-S24 regarding TLR 2, 4 and 7 (p<0.05) as well as between S72-S48 and S72-S24 for all TLRs examined (p<0.05).

Besides examination of the lung, we further examined the expression of TLRs in the intestinal tissues by means of qRT-PCR. mRNA levels of TLRs 2, 3, 4 and 7 were significantly higher in all S-groups compared to their respective C-groups (p<0.05), except for S24-C24 regarding TLRs 2 and 3. Among S-groups, significant differences were observed between S48-S24 regarding TLR 2, 4 and 7 (p<0.05) as well as between S72-S48 and S72-S24 for all TLRs examined (p<0.05) (Fig 4). No statistical differences were found between S48 and S24 groups concerning TLR 3 (S4 Table).

Eventually, we evaluated the differential expression of all TLRs in the intestine and lung (S5 Table). Correlations were extracted comparing intestinal and lung tissues from time-adjusted sepsis groups. Lung tissues showed a significantly higher expression of TLR 2 and 4 compared to the intestinal tissues at 24, 48 and 72 hours (p<0.05), whereas TLR 3 was higher expressed in the intestine at all time points examined (p<0.05). TLR 7 levels were significantly higher in the intestinal tissues at 24 hours (p<0.0001), while lungs predominated at 48 hours (p<0.0001). Finally, no significant difference was found between lung and intestine at 72 hours with regards to TLR 7 expression.

## Discussion

Our study showed that TLR 2, 3, 4 and 7 were highly expressed in the lungs of septic mice, while the same receptors were only slightly expressed in controls. Interestingly, statistical differences were observed between time-adjusted S- and C-groups (S24-C24, S48-C48 and S72-C72) regarding the expression of every TLR, with the highest differences being noticed between S72-C72 concerning TLR 2 and TLR 4. Furthermore, it was clear that the expression of each TLR type gradually increased among sepsis groups as time to euthanasia was longer.

Similarly, expression of TLRs in the intestines of septic mice showed a gradual increase from 24 to 72 hours until euthanasia. Again, sepsis groups yielded a significantly higher expression of all TLRs compared to the control groups. Statistical differences were not reached only between S24-C24 with regards to TLR 2 and 3. It is, therefore, obvious that the alterations of TLRs expression under investigation were in the same line with regards to two different systems; respiratory and gastrointestinal system. The above-mentioned findings may suggest that TLRs are implicated in the alterations of lung physiology during sepsis.

Developing an animal model is essential for the detailed understanding of the complex mechanisms that occur during sepsis. In brief, CLP procedure involves three insults; a. trauma by means of laparotomy, b. necrosis caused by ligation of the cecum and c. infection due to the leakage of peritoneal microbial flora into the peritoneum [9]. It has been largely popularized mainly due to certain characteristics; a. simplicity of the procedure, b. polymicrobial infection, c. presence of an infectious focus, d. pathogens originating from the host, e. presence of both hyper- and hypo-inflammatory phases and f. prolonged and lower elevation of cytokine release as in humans [9,11,21].

Although CLP is a well-established method considered to closely resemble human response [9,11,22], the results produced in the preclinical setting should not arbitrarily be extrapolated to the humans. Multiple factors such as the genetic background, gender, age, immune and nutritional status, supportive care and other pre-existing comorbidities may influence the outcomes of sepsis in humans [10]. Laboratory mice are more homogeneous with respect to these characteristics than real life septic patients [11]. Of note, age is a strong determinant of the clinical outcomes. Preclinical data have demonstrated the positive relationship of age with mortality rates in sepsis mouse models [23]. In our study, we used animals aged 12–14 weeks that are equivalent of around 10–11 years of age in humans. This should be kept in mind when interpreting these results given that sepsis predominantly occurs after the age of 50 in the clinical setting.

Several factors affect the severity of bacterial sepsis in the CLP model, including the length of the ligated cecum, the circadian rhythm (the time of the day sepsis is induced), sex, age and strain of the animals, heterogeneity of the animal host response, supporting treatment to the animal (i.e. fluid resuscitation and administration of antibiotics) as well as the surgical skills of the operating person [11]. Since the position of cecal ligation is a major determinant of sepsis severity and mortality [24], ligation was performed at the distal one-third of the cecum producing mid-grade sepsis [18]. Moreover, in order to limit the effect of individual’s surgical skills on the severity of bacterial sepsis, all operations were executed by the same person in accordance to a previous published protocol [18]. In addition, the diameter of the needle used for the perforation as well as the number of punctures are two key factors that determine the amount of microbial dose entering the peritoneum [21]. In our experiments, we constantly used needles of intermediate diameter, such as 21-gauge. Additionally, in the piloting setting, we observed that one puncture was enough to induce peritonitis and sepsis resulting in death for the majority of the septic mice on the 4^th^ postoperative day, whereas more penetrations resulted in early death, sometimes within 24 hours of onset. Therefore, we insisted on the first option.

In our study, the severity of CLP-induced sepsis in each mice group was assessed based on criteria proposed by Doerflinger et al [19]. A scoring system comprising of parameters, such as appearance, breathing pattern, weight change, behavior and provoked reaction was utilized [19] along with the changes in cytokine and biochemical marker levels. Following standardization of the protocol in the piloting setting, all animals fulfilled the criteria of sepsis and were included in our study. Interestingly, sepsis groups demonstrated a significant elevation in creatinine, IL-6 and IL-10 when compared to hour-adjusted control groups, thus indicating a possible dysfunction of more organs including the kidneys. In addition, the RBC count showed a gradual decline during the course of sepsis. Two mechanisms may be implicated; a. bone marrow suppression in the context of sepsis and b. hemolysis induced by the rapid increase in RBC sphericity, decreased deformability and thus destruction [25].

To date, the exact mechanisms of sepsis-induced lung injury are not completely understood. It is known, though, that the innate immune system first reacts to pathogen-associated molecular patterns (PAMPs) through activating pattern recognition receptors (PRRs) such as TLRs [26]. Those molecules aid in recognizing septic products as well as initiating inflammatory responses. TLR 4 typically responds to gram-negative bacteria, whereas TLR 2 mainly senses gram-positive. Furthermore, TLR 3 and TLR 7 seem to primarily react to invading viruses [27,28]. In humans, TLRs are expressed in bronchial or endothelial lung cells as well as infiltrating cells of myeloid or lymphoid origin, such as alveolar macrophages, neutrophils and dendritic cells. As a result, these receptors contribute to the development of ALI or ARDS either from infectious or non-infectious causes [28].

Our study suggests a distant effect phenomenon. Cecal puncture led to the development of peritonitis and sepsis, which seems to have further affected the respiratory system. Although several mechanisms have been recommended, the exact pathway of inducing ALI/ARDS has not been determined yet. However, Dickson et al. have recently identified gut microbiota in the lungs of ARDS patients, thus proposing translocation of gut bacteria to the lungs as a shared mechanism between sepsis and ARDS [29]. This is in accordance with our findings, since TLRs, known as recognizing microbes, were highly expressed in the lungs of septic mice.

At this point, it would be tempting to note that intervention in the cascade of sepsis by inhibiting TLRs could perhaps prove beneficial in arresting the development of sepsis and could, therefore, result in more favorable clinical outcomes.

## Conclusion

TLRs seem to be implicated in ARDS pathogenesis during sepsis. While more studies need to be conducted in order to completely understand the nature of TLRs and ligands involved, this family of receptors may represent a promising target for establishing novel therapeutic strategies in the treatment of sepsis.

## Supporting information

S1 Table Primers that were used for PCR. (PDF)

S2 Table Changes of TLR 2, 3, 4 and 7 in immunofluorescence analysis at the experimental time points. Values are expressed as the mean±SD. Statistical significances (*p*<0.05) between the groups at the same time point are indicated as follows: a: Control (C) vs Septic (S); b: 24S vs 48S; c: 48S vs 72S: d: 24S vs 72S.
(PDF)

S3 Table PCR showing changes of TLR 2, 3, 4 and 7 in lung tissues at the experimental time points. Values are expressed as the mean±SD. Statistical significances (*p*<0.05) between the groups at the same time point are indicated as follows: a Control (C) vs Septic (S); b: 24S vs 48S; c: 48S vs 72S: d: 24S vs 72S.
(PDF)

S4 Table PCR showing changes of TLR 2, 3, 4 and 7 in the intestine at the experimental time points. Values are expressed as the mean±SD. Statistical significances (*p*<0.05) between the groups at the same time point are indicated as follows: a Control (C) vs Septic (S); b: 24S vs 48S; c: 48S vs 72S: d: 24S vs 72S.
(PDF)

S5 Table Differential expression of all TLRs in the intestine and lung among time-adjusted sepsis groups. The alpha level of statistical significance was set at 0.05. *p-value denoting higher expression in the lung compared to the intestine. ^§^p-value denoting higher expression in the intestine compared to the lung.
(PDF)

S6 Table The ARRIVE guidelines checklist. (PDF)

S7 Table Minimal data set for analyses. (PDF)

S8 Table Minimal data set for analyses. (PDF)

S9 Table Minimal data set for analyses. (PDF)
