# Supplementary material for: Expression of Toll-like receptors (TLRs) in the lungs of an experimental sepsis mouse model
Source: PLoS One. 2017 Nov 14;12(11):e0188050. doi: 10.1371/journal.pone.0188050 (PMC5685586; doi:10.1371/journal.pone.0188050)
Supplement: S1 Table — (PDF) [file pone.0188050.s001.pdf]

TLR's / Primer	TLR-2	TLR-3	TLR-4	TLR-7
LEFT	5'-tgggctgacttctctcaatg-3'	5'-ctgggtctgggaacatttct-3'	5'-ggaacaaacagcctgagaca-3'	5'-atacctggccactgatgtga-3'
TM	59,0	59,0	58,9	59,0
RIGHT	5'-ttcatcggtgagctgacttc-3'	5'-ttgctgaactgcgtgatgta-3'	5'-ttgagactggtcaagccaag-3'	5'-gactccatggattgcagatg-3'
TM	59,0	59,0	59,0	59,0
Product Size	243	211	233	249
NCBI reference sequence	NM 011905	NM 126166	NM 021297.2	NM 133211.3

Supplemental Table 1. Primers that were used for PCR
