# Supplementary material for: Expression of Toll-like receptors (TLRs) in the lungs of an experimental sepsis mouse model
Source: PLoS One. 2017 Nov 14;12(11):e0188050. doi: 10.1371/journal.pone.0188050 (PMC5685586; doi:10.1371/journal.pone.0188050)
Supplement: S2 Table — Values are expressed as the mean±SD. Statistical significances (p<0.05) between the groups at the same time point are indicated as follows: a: Control (C) vs Septic (S); b: 24S vs 48S; c: 48S vs 72S: d: 24S vs 72S. (PDF) [file pone.0188050.s002.pdf]

Time points<br><br>Groups	24h	48h	72h
TLR-2			
Control	-[a]	-[a]	-[a]
Septic	0.33±0.21	1.76±0.5[b]	3.36±0.34[c, d]
TLR-3			
Control	-[a]	-[a]	-[a]
Septic	0.16±0.1	1.27±0.33[b]	2.24±0.19[c, d]
TLR-4			
Control	-[a]	-[a]	-[a]
Septic	0.26±0.25	0.58±0.28[b]	1.11±0.04[c, d]
TLR-7			
Control	-[a]	-[a]	-[a]
Septic	0.33±0.17	1.52±0.4[b]	1.86±0.64 [d]

Supplemental table 2. Changes of TLR-2, 3, 4 and 7 in immunofluorescence analysis at the experimental time points. Values are expressed as the mean±SD. Statistical significances (p<0.05) between the groups at the same time point are indicated as follows: a: Control (C) vs Septic (S); b: 24S vs 48S; c: 48S vs 72S: d: 24S vs 72S.
