# Supplementary material for: Expression of Toll-like receptors (TLRs) in the lungs of an experimental sepsis mouse model
Source: PLoS One. 2017 Nov 14;12(11):e0188050. doi: 10.1371/journal.pone.0188050 (PMC5685586; doi:10.1371/journal.pone.0188050)
Supplement: S3 Table — Values are expressed as the mean±SD. Statistical significances (p<0.05) between the groups at the same time point are indicated as follows: a Control (C) vs Septic (S); b: 24S vs 48S; c: 48S vs 72S: d: 24S vs 72S. (PDF) [file pone.0188050.s003.pdf]

Time points<br><br>Groups	24h	48h	72h
TLR-2			
Control	-[a]	-[a]	-[a]
Septic	23.20±5.76	62.9±0.55[b]	110.7±0.82[c, d]
TLR-3			
Control	-[a]	-[a]	-[a]
Septic	2.85±0.36	5.08±0.77[b]	17.58±0.37[c, d]
TLR-4			
Control	-[a]	-[a]	-[a]
Septic	21.64±0.39	76.16±1.15[b]	112.2±1.78[c, d]
TLR-7			
Control	-[a]	-[a]	-[a]
Septic	5.64±0.54	45.93±0.82[b]	50.71±0.5[c, d]

Supplemental table 3. PCR showing changes of TLR 2, 3, 4 and 7 in lung tissues at the experimental time points. Values are expressed as the mean±SD. Statistical significances (p<0.05) between the groups at the same time point are indicated as follows: a Control (C) vs Septic (S); b: 24S vs 48S; c: 48S vs 72S: d: 24S vs 72S.
