# Supplementary material for: Expression of Toll-like receptors (TLRs) in the lungs of an experimental sepsis mouse model
Source: PLoS One. 2017 Nov 14;12(11):e0188050. doi: 10.1371/journal.pone.0188050 (PMC5685586; doi:10.1371/journal.pone.0188050)
Supplement: S4 Table — Values are expressed as the mean±SD. Statistical significances (p<0.05) between the groups at the same time point are indicated as follows: a Control (C) vs Septic (S); b: 24S vs 48S; c: 48S vs 72S: d: 24S vs 72S. (PDF) [file pone.0188050.s004.pdf]

**Time points**<br><br>**Groups**	**24h**	**48h**	**72h**
TLR-2			
Control	-	-[a]	-[a]
Septic	4.86±0.53	15.79±0.49[b]	79.76±6.38[c, d]
TLR-3			
Control	-	-[a]	-[a]
Septic	4.12±0.13	7.75±0.3	31±4.7[c, d]
TLR-4			
Control	-[a]	-[a]	-[a]
Septic	7.16±0.86	15.06±0.58[b]	75.19±2.31[c, d]
TLR-7			
Control	-[a]	-[a]	-[a]
Septic	22.5±1.1	32.8±0.25[b]	55.63±4.87[c, d]

Supplemental table 4. PCR showing changes of TLR 2, 3, 4 and 7 in the intestine at the experimental time points. Values are expressed as the mean±SD. Statistical significances (p<0.05) between the groups at the same time point are indicated as follows: a Control (C) vs Septic (S); b: 24S vs 48S; c: 48S vs 72S: d: 24S vs 72S.
