# Supplementary material for: Expression of Toll-like receptors (TLRs) in the lungs of an experimental sepsis mouse model
Source: PLoS One. 2017 Nov 14;12(11):e0188050. doi: 10.1371/journal.pone.0188050 (PMC5685586; doi:10.1371/journal.pone.0188050)
Supplement: S5 Table — The alpha level of statistical significance was set at 0.05. *p-value denoting higher expression in the lung compared to the intestine. §p-value denoting higher expression in the intestine compared to the lung. (PDF) [file pone.0188050.s005.pdf]

**Time points**	**24h**	**48h**	**72h**
TLR2	0,0054*	<0.0001*	0,0011*
TLR3	0,0047§	0,0051§	0,0078§
TLR4	<0.0001*	<0.0001*	<0.0001*
TLR7	<0.0001§	<0.0001*	0,1573

Supplemental table 5 Differential expression of all TLRs in the intestine and lung among time-adjusted sepsis groups. The alpha level of statistical significance was set at 0.05.

*p-value denoting higher expression in the lung compared to the intestine

§p-value denoting higher expression in the intestine compared to the lung
