# Supplementary material for: Expression of Toll-like receptors (TLRs) in the lungs of an experimental sepsis mouse model
Source: PLoS One. 2017 Nov 14;12(11):e0188050. doi: 10.1371/journal.pone.0188050 (PMC5685586; doi:10.1371/journal.pone.0188050)
Supplement: S7 Table — (PDF) [file pone.0188050.s007.pdf]

Supplemental Table 7 Minimal data set-Blood and biochemical results

WBC	24C	24S	48C	48S	72C	72S
N=3	7,3	2,6	9,5	3,3	9,7	5,9
	5,71	1,6	8,69	3,4	8,5	5,56
	6,8	2,4	4,49	3,9	5,85	5,1

RBC	24C	24S	48C	48S	72C	72S
N=3	5,69	4,65	9,96	6,25	8,44	1,69
	5,87	5,12	7,12	7,89	9,28	1,85
	6,59	6,58	7	5,2	7,02	1,69

LY	24C	24S	48C	48S	72C	72S
N=3	71	59	81	52	68	32
	68	58	68	55	86	38
	79	65	78	56	74	32

MO	24C	24S	48C	48S	72C	72S
N=3	2	2	2	5	2	7
	2	5	5	5	6	8
	3	6	6	6	3	7

CR	24C	24S	48C	48S	72C	72S
N=3	0,39	1,66	0,26	2,28	0,36	5,18
	0,12	1,34	0,29	2,92	0,54	3,96
	0,56	1,76	0,64	2,56	0,12	3,25

IL6	24C	24S	48C	48S	72C	72S
N=3	10,1	1120	11,25	2152	19,45	2598
	11	1238	10,36	3651	12,85	2895
	12,36	1450	10,84	2436	13,25	2259

IL10	24C	24S	48C	48S	72C	72S
N=3	7,15	104	9,58	150,25	9,25	495
	7,1	86	7,85	221	8,47	589
	6,23	85	6,58	188,56	6,58	1789
