# Supplementary material for: Expression of Toll-like receptors (TLRs) in the lungs of an experimental sepsis mouse model
Source: PLoS One. 2017 Nov 14;12(11):e0188050. doi: 10.1371/journal.pone.0188050 (PMC5685586; doi:10.1371/journal.pone.0188050)
Supplement: S8 Table — (PDF) [file pone.0188050.s008.pdf]

Supplemental Table 8 Minimal data set- IHC for septic groups

AREA %

TLR2	N=3	S24	S48	S72
IHC		0,28	1,45	3,11
		0,12	1,69	3,79
		0,34	1,14	3,8
TLR3	N=3	S24	S48	S72
IHC		0,24	1,02	2,1
		0,28	1,9	2,14
		0,21	1,09	2,62
		S24	S48	S72
TLR4	N=3	0,06	0,64	1,15
IHC		0,05	0,68	1,05
		0,37	0,22	1,15
TLR7	N=3	S24	S48	S72
IHC		0,64	1,69	2,63
		0,29	1,09	2,12
		0,25	1,5	1,12
