# Supplementary material for: Expression of Toll-like receptors (TLRs) in the lungs of an experimental sepsis mouse model
Source: PLoS One. 2017 Nov 14;12(11):e0188050. doi: 10.1371/journal.pone.0188050 (PMC5685586; doi:10.1371/journal.pone.0188050)
Supplement: S9 Table — (PDF) [file pone.0188050.s009.pdf]

Supplemental Table 9 Minimal data set-PCR for septic groups

TLR2	S24	S48	S72
N=3	19,62	62,35	110
	19,24	61,2	86,3
	23,26	65,3	99,5
TLR3	S24	S48	S72
N=3	3,14	5,23	17,52
	2,12	4,02	16,23
	2,09	5,26	14,26
TLR4	S24	S48	S72
N=3	21,36	77,45	110,25
	19,56	42,36	96,32
	20,47	71,56	102,89
TLR7	S24	S48	S72
N=3	5,26	45,69	50,26
	6,02	40,12	48,26
	5,12	39,63	46,2
